# Comprehensive Curation and Harmonization of Small-Molecule MS/MS Libraries in Spectraverse

**DOI:** 10.1021/acs.analchem.5c06256

**Published:** 2026-01-26

**Authors:** Vishu Gupta, Hantao Qiang, Hsin-Hsiang Chung, Ehud Herbst, Michael A. Skinnider

**Affiliations:** † Lewis-Sigler Institute for Integrative Genomics, 6740 Princeton University, Princeton, New Jersey 08544, United States; ‡ Ludwig Institute for Cancer Research, 6740 Princeton University, Princeton, New Jersey 08544, United States; § Department of Chemistry, 6740 Princeton University, Princeton, New Jersey 08544, United States

## Abstract

Reference libraries of tandem mass spectra (MS/MS) are widely used for metabolite identification in untargeted metabolomics and to train machine-learning models for metabolite annotation. However, public spectral libraries are scattered across disparate databases and contain spectra that are of low resolution or quality, missing critical metadata, or which have chemically incoherent annotations. Addressing these issues requires extensive preprocessing and considerable expertise in mass spectrometry, which presents a significant barrier to investigators interested in developing their own machine-learning models. Here, we present Spectraverse, a comprehensive and extensively curated library of public MS/MS spectra from small molecules. We assembled reference spectra from both major repositories and previously overlooked resources and then developed a preprocessing pipeline to harmonize metadata, standardize chemical structures, and remove low-quality or redundant spectra. These efforts led us to identify previously undocumented pitfalls in existing public libraries that may have confounded prior comparisons of machine-learning models or conversely have caused valid MS/MS spectra to have been discarded from the training sets of these models. The resulting resource affords the most comprehensive coverage of chemical space of any machine-learning-ready library of MS/MS spectra to date while also expanding the coverage of adducts and ionization modes encountered in metabolomics experiments. We intend to maintain and expand Spectraverse in order to encompass the growing number of publicly available reference MS/MS spectra that can be expected to accumulate in the future.

## Graphical abstract

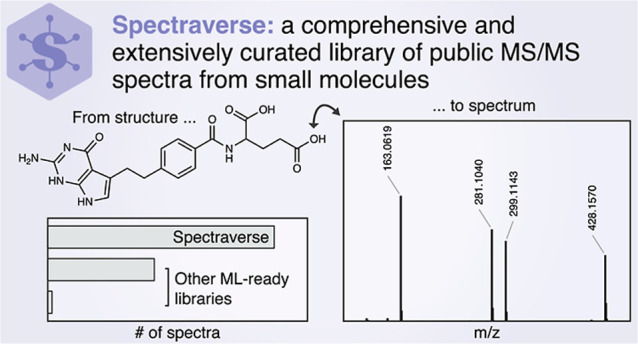

## Introduction

Mass spectrometry-based metabolomics routinely detects thousands of small-molecule-associated signals within biological samples.^1^ Tandem mass spectrometry (MS/MS) is a key technique by which these signals are linked to the chemical structures of the corresponding small molecules.^2,3^ MS/MS spectra acquired in an untargeted metabolomics experiment are often annotated via comparisons to libraries of reference MS/MS spectra.^4–7^ Typically, however, only a fraction of MS/MS spectra can be annotated through spectral library search, in part because these libraries are limited in size and are generally biased toward well-studied, commercially available compounds.^8,9^ To address this gap, computational interpretation of MS/MS spectra has emerged as an important research direction in metabolomics.^10,11^ Over the past few years, a series of increasingly sophisticated machine-learning approaches have been introduced that attempt to identify small molecules from their MS/MS spectra, even when these small molecules do not appear in any reference spectral library.^12–29^ Such approaches generally either aim to generate in silico libraries of predicted MS/MS spectra (so-called “compound-to-MS/MS” approaches)^13–17,19,26,28–30^ or to predict molecular descriptors from MS/MS spectra themselves (so-called “MS/MS-to-compound” approaches).^12,20,21,27^ As with other supervised machine-learning tasks, the success of these approaches is contingent on the availability of large, diverse, and high-quality training data sets—in this case, reference libraries of MS/MS spectra annotated with the chemical structures of the corresponding small molecules.

Reference MS/MS libraries thus play a central role in both the analysis of metabolomic experiments and the development of computational methods for MS/MS interpretation. In practice, however, there is little consensus on which library to use for these purposes. Commercial spectral libraries (such as NIST, mzVault, or METLIN) generally offer well-curated and annotated MS/MS spectra, but these libraries are often not provided in a format that can be used to train machine-learning models (for instance, because the underlying spectra and their associated metadata cannot be exported from proprietary software). Moreover, because these libraries cannot be redistributed, machine-learning models trained on commercial libraries cannot readily be reproduced.^31^ To circumvent these issues, several groups have instead leveraged publicly available reference spectral libraries to train machine-learning models, such as those aggregated by GNPS or MoNA.^12,17,18,20,22–25,27,29,30^ However, because public spectral libraries generally have not been subjected to the same degree of curation as commercial libraries, developers must formulate criteria to preprocess these spectra, identify high-quality spectra, and verify the integrity of the associated metadata. Moreover, public spectral libraries are scattered across disparate databases. Although it has become increasingly common to aggregate spectra from multiple sources, machine-learning models for MS/MS interpretation have historically been trained on individual (often very small) data sets. It has been appreciated for several decades that differences in the data sets used to train various machine-learning models have hindered fair comparisons between them.^32^ The use of relatively small training data sets may have also led the performance of some approaches to be underestimated, an issue that could in principle be remedied given that hundreds of thousands of MS/MS spectra are now available for model training.

Here, we present Spectraverse, a comprehensive and extensively curated resource of public MS/MS spectra for metabolite annotation and machine-learning model development. To assemble Spectraverse, we sought to identify all publicly available reference spectra, including not just those already included in public databases such as GNPS and MoNA but also libraries reported in the literature that had never been deposited in such databases. We devised and implemented an extensive preprocessing pipeline to identify high-quality MS/MS spectra, curate and harmonize their associated metadata, and standardize the representations of their associated chemical structures (Figure 1). In the course of these efforts, we identified a series of pitfalls in publicly available spectral libraries that do not appear to have previously been documented, which we describe here. Spectraverse expands on the largest existing collection of public MS/MS spectra that has been used to train and benchmark machine-learning models, MassSpecGym, by almost 250,000 spectra and should provide a foundation for metabolite annotation and open model development. Spectraverse contains 488,630 MS/MS spectra from 44,237 unique small molecules and is freely available from Zenodo at https://doi.org/10.5281/zenodo.17870921.

**Figure 1:**
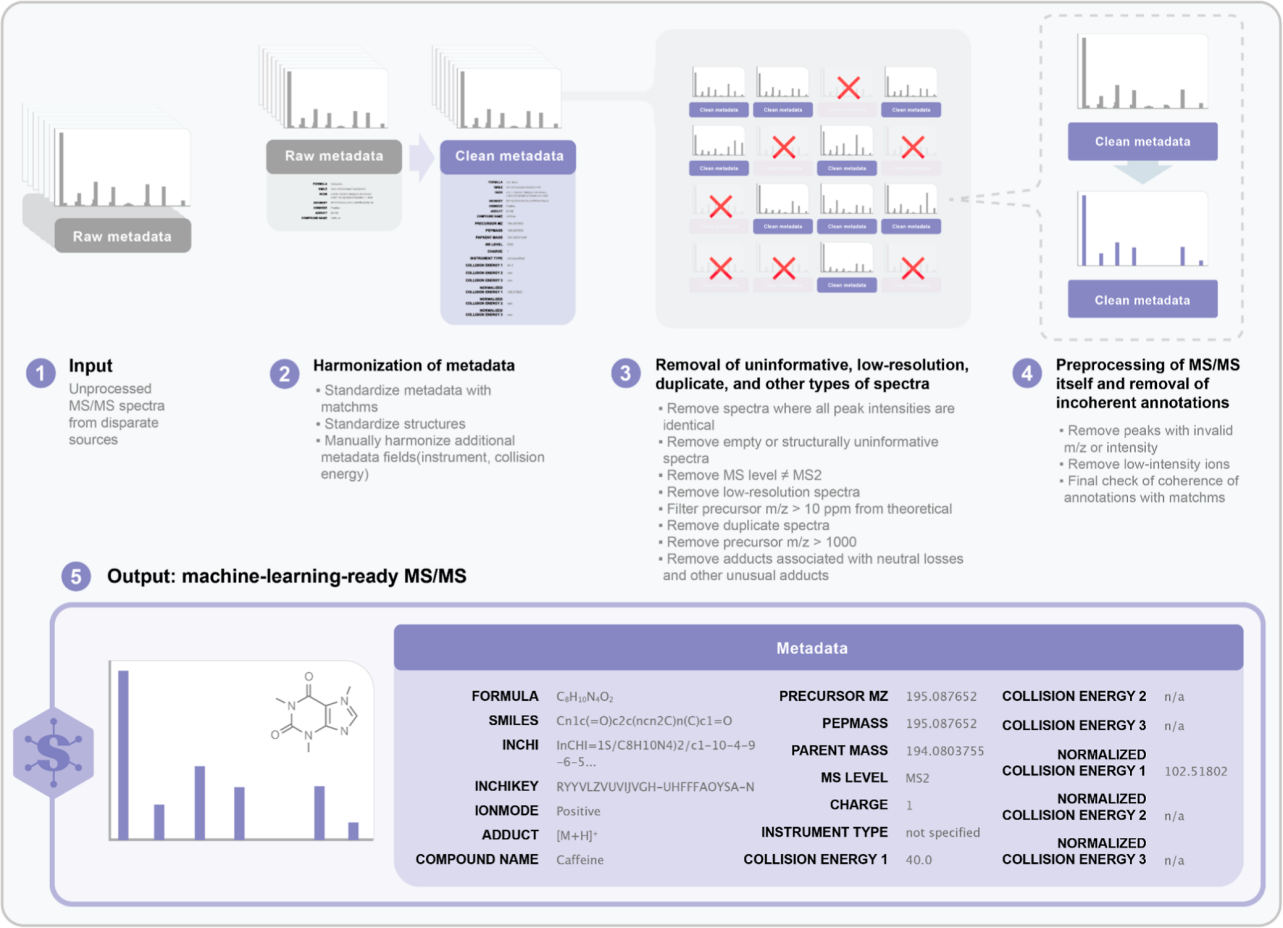
Schematic overview of the major steps for harmonization and preprocessing of MS/MS spectra and their associated metadata in Spectraverse.

## Methods

### Data Sources

We began by assembling a comprehensive collection of publicly available reference MS/MS spectra. In brief, we first retrieved reference MS/MS libraries from a series of major databases and community resources, including GNPS, MoNA, MS-DIAL,^33^ HMDB,^34^ and MSnLib.^35^ We then supplemented these with reference MS/MS libraries that had been described in individual publications but which to our knowledge had not been deposited to databases such as GNPS or MoNA.^36–45^ Spectral libraries were obtained from the Supporting Information of these publications, by reprocessing raw experimental data deposited to repositories such as MetaboLights,^46^ or from GitHub repositories, Zenodo accessions, or Web sites accompanying the publications themselves; data collection is described in detail in the Supporting Information. A total of 1,672,217 MS/MS spectra were retrieved and carried forward for further preprocessing in MGF format, as described below. In general, when the optimal means of preprocessing the data could not be unambiguously determined, we favored more permissive approaches to preprocessing so as to allow for the optional application of more stringent filters by future users of Spectraverse.

### Metadata Standardization and Repair

We first standardized the names and values of key metadata fields. For instance, information about the adduct associated with a given spectrum was sometimes found in fields such as MODIFICATIONS or PRECURSOR_TYPE; these were standardized so that adduct information was always stored in the ADDUCT field. Analogously, ionization modes were standardized by recoding “p” or “POS” to “positive”. Similar adjustments were made to ensure that compound names, SMILES, InChIs and InChIKeys, charges, and MS levels were each assigned in a consistent metadata field and, for categorical metadata, with consistent values.

Structures for spectra that were not associated with a compound name or structural identifier were retrieved by querying the GNPS API when possible, and we attempted to manually curate SMILES strings for many of the remaining spectra not associated with a chemical structure, focusing our efforts, in particular, on compound names that were associated with multiple MS/MS spectra. When a spectrum was associated with an invalid SMILES string, we tested whether a valid SMILES could be derived by modifications to the string (for instance, via the removal of extraneous characters).

At this stage, fragment ions with zero intensity were removed, as were ions outside the *m*/*z* range from 10 to 1000.

Following manual curation of the metadata associated with each MS/MS spectrum, we applied matchms^31^ (version 0.27.0) to further harmonize and repair metadata, where possible. In particular, matchms was used to harmonize metadata field names and values, derive missing metadata or metadata entered in incorrect fields, and repair annotations; the specific configuration with which matchms was run is provided in the Supporting Information. Fragment intensities were also normalized to the base peak at this stage. The manual standardization described above was required prior to the use of matchms in order to prevent the erroneous removal of spectra with unusually coded metadata fields or values. Following this first matchms run, we also removed spectra with uniform peak intensities, with precursor *m*/*z* values greater than 1,000, and spectra that were still not associated with a valid SMILES string. Specific filters applied in the first matchms run were as follows:

- HARMONIZE_METADATA_FIELD_NAMES: charge values were converted to integers, compound names were standardized, and precursor *m*/*z* values were assigned when they were indicated in the PEPMASS field.
- DERIVE_METADATA_IN_WRONG_FIELD: adduct and molecular formula information incorrectly embedded in compound names were extracted. Compound names were cleaned, ionization modes were inferred, and inconsistent or missing InChI, InChIKey, and SMILES representations were repaired.
- HARMONIZE_METADATA_ENTRIES: undefined InChIKey, InChI, and SMILES were standardized across the data set. Adduct information was cleaned and uniformly formatted.
- DERIVE_MISSING_METADATA: missing metadata were derived where possible, including correcting charge, estimating parent mass, generating InChI from SMILES, SMILES from InChI, InChIKey from InChI, and molecular formula from SMILES.
- REPAIR_ANNOTATION: SMILES containing certain salts were corrected (repair_smiles_of_salts), parent masses were recalculated from SMILES (repair_parent_mass_from_smiles) or adjusted if incorrectly assigned as molar mass (repair_parent_mass_is_molar_mass), and adducts were corrected by comparing parent masses and precursor *m*/*z* values (repair_adduct_based_on_parent_mass). Mismatching structure identifiers were repaired by selecting the SMILES or InChI matching the parent mass (repair_not_matching_annotation), and missing structural metadata (SMILES, InChI, or InChIKey) was derived from compound names (derive_annotation_from_compound_name). We modified the code in get_neutral_mass_from_smiles.py (function _get_neutral_mass) to ignore spectra annotated with [M]+ or [M]– adducts that had a reported charge of zero.
- NORMALIZE_INTENSITY: fragment ion intensities were scaled to the range (0, 1] by normalizing to the base peak.

### Standardization of Chemical Structures

We devised a multistage approach to preprocess and standardize chemical structures. SMILES strings were loaded in RDKit, sanitized, and cleaned to remove hydrogens, metals, and disconnected fragments using the RDKit functions SanitizeMol, Cleanup, and FragmentParent, respectively. Stereochemical information was removed, and tautomers were standardized to their canonical forms using the TautomerEnumerator class. Charges were neutralized whenever possible. We found that our approach sometimes incorrectly neutralized functional groups that were written in charge-separated forms, notably sulfoxides and phosphoryl groups, and therefore, we implemented additional checks to ensure that these functional groups were correctly neutralized. When neutralization altered the overall charge of the molecule, two copies of the spectrum were created (one associated with the charged form and one with the uncharged form), and both were brought forward to the second round of matchms described below, which we found to be necessary in order to prevent matchms from removing zwitterionic compounds for which the process of neutralizing charges yielded a charged molecule. At this stage, empty spectra, non-MS2 spectra, and spectra from rare adducts (i.e., adducts other than the nine common adducts listed below, none of which were individually associated with more than approximately 5000 spectra after the first matchms run) or adducts associated with neutral losses were removed.

### Removal of Low-Quality or Incoherently Annotated Spectra

We then sought to identify and remove spectra that were inferred to be of low quality or resolution or which were annotated with chemical structures that were inconsistent with the spectra themselves. First, we removed low-resolution spectra in which all fragment masses were written to two or fewer decimal places. We removed spectra for which all fragment *m*/*z* values exceeded the precursor *m*/*z*, as well as spectra containing only one fragment ion when this fragment was within ±1.6 *m*/*z* of the precursor *m*/*z*. The spectra were then subjected to a second round of matchms to remove spectra with incoherent annotations. The code in the matchms function _get_neutral_mass was modified in order to remove spectra that were annotated as [M]^+^ or [M]^−^ adducts but which had a net charge of zero. In addition to all of the filters from the first matchms run, the following additional filters were applied:

- REQUIRE_COMPLETE_METADATA: spectra were retained only if they had valid precursor *m*/*z* values (require_precursor_mz) and a valid ionization mode (require_correct_ionmode, positive or negative).
- REQUIRE_COMPLETE_ANNOTATION: spectra were filtered based on the consistency of the associated structural metadata. In particular, the parent mass had to match the calculated monoisotopic mass of the structure encoded in the SMILES field to within 0.1 Da (require_parent_mass_match_smiles), annotations had to be valid (require_valid_annotation), the theoretical *m*/*z* of the adduct had to match the precursor *m*/*z* within 0.1 Da (require_matching_adduct_precursor_mz_parent_mass), and the adduct had to match the annotated ionization mode (require_matching_adduct_and_ionmode).

### Removal of Near-Duplicate Spectra

Candidate pairs of near-duplicate spectra were identified by matching spectra based on the first 14 characters of the InChIKey and polarity. The cosine similarity was then calculated between each of these candidate pairs, and spectra with cosine similarities exceeding 0.99 were flagged as near-duplicates. A single entry from each set of near-duplicates was retained, favoring entries with greater numbers of fragment ions when possible.

### Final Filters and Metadata Standardization

A final check for high-resolution MS/MS spectra was implemented by removing spectra for which the deviation between the theoretical and experimental precursor *m*/*z* values exceeded 10 ppm. Rare adducts that had been introduced by the second round of matchms were removed such that the nine most common adducts were retained in Spectraverse ([M + H]^+^, [M + Na]^+^, [M + K]^+^, [M + NH_4_]^+^, [M]^+^, [M – H]^−^, [M + Cl]^−^, [M + HCOOH – H]^−^, and [M + CH_3_COOH – H]^−^). Fragment ions with relative intensities below 0.1% were removed, and only the top-4096 highest intensity ions per spectrum were retained. A handful of spectra annotated with a SMILES string containing radical electrons were removed at this stage.

Last, we standardized metadata associated with instrument type and collision energy. Instrument types were manually mapped to three categories: QTOF, Orbitrap, and ion trap (or not specified). At this stage, spectra from low-resolution triple quadrupole (QQQ) instruments were removed. To accommodate ramped or stepped collision energies, each spectrum was associated with up to three collision energy fields, each of which is provided both in electron volts (eV) and as a normalized collision energy (NCE) within the spectral metadata. In particular, ramped CE values were assigned to the fields CE1 and CE2 (NCE1 and NCE2), whereas stepped CE/NCE values were assigned to CE1, CE2, and CE3 (or NCE1/NCE2/NCE3).

### Centroiding, Electronic Denoising, and Removal of Low-Intensity Fragments

Electronic denoising^47^ was performed using a function implemented in the “spectral_denoising” Python package (https://github.com/FanzhouKong/spectral_denoising). Centroiding was performed using a custom function adapted from the “MSEntropy” Python package (https://github.com/YuanyueLi/MSEntropy),^47^ which grouped peaks within a prespecified *m*/*z* tolerance range and calculated a weighted average *m*/*z* for each peak group. To assess the effectiveness of centroiding, electronic denoising, and removal of low-intensity fragments, we evaluated the propensity for spectral similarity (as quantified by the cosine similarity) to correctly differentiate spectra from the same versus different compounds, as quantified by the area under the receiver operating characteristic curve (AUROC). Pairwise cosine similarities between spectra were computed to generate a similarity matrix, considering only spectrum pairs with precursor *m*/*z* values within a 10 ppm window. In parallel, a binary label matrix was created, assigning a value of 1 to spectra sharing the same first 14 characters of their InChIKeys, and 0 otherwise. The AUROC was then calculated with the roc_auc_score function from the sklearn library. For centroiding, we considered various different *m*/*z* tolerance thresholds to merge peaks and also considered applying our centroiding function only to spectra containing a certain minimum number of fragment ions. In parallel, we evaluated the impact of retaining only the top-*n* highest intensity fragment ions per spectrum. We found that centroiding and denoising both modestly but consistently decreased the AUROC and therefore elected not to include either function in our preprocessing pipeline. Removing low-intensity ions likewise modestly decreased the AUROC, but this effect had largely saturated when retaining the top-4096 highest intensity fragment ions, and as a result, we applied this lenient filter in Spectraverse.

### Comparison to MassSpecGym and NPLIB1

We compared Spectraverse to MassSpecGym and NPLIB1, the two public spectral libraries that have been the most widely used to train and benchmark machine-learning models. MassSpecGym is a recently proposed benchmark data set for the computational interpretation of MS/MS spectra from small molecules.^48^ Spectra were obtained from HuggingFace (file MassSpecGym.mgf), and no further processing was applied. NPLIB1 ^20^ comprises reference MS/MS spectra extracted from GNPS that were prepared by the authors of CANOPUS.^49^ Spectra were obtained from Zenodo (doi: 10.5281/zenodo.8316682, file canopus_train_export_v2.tar), following instructions in the MIST GitHub repository. The resulting archive contained 10,709 “.ms” files, in line with the size of the data set as described in prior work.^17,20,30,50–52^ Some of these files contained multiple spectra, which were merged using the function “combineSpectra” in the Bioconductor package “Spectra” with a tolerance of 50 ppm; spectra whose headers indicated they were MS1 spectra were removed prior to merging. UMAP was run on the union of unique chemical structures found in all three data sets, using the implementation in the “umap-learn” Python package, with the number of neighbors (n_neighbors) set to 5000 and the minimum distance (min_dist) set to 0.5, and was visualized as a 2D density plot. ClassyFire annotations were obtained from the ClassyFire API using the classyfireR package (https://github.com/aberHRML/classyfireR).^53^ The coverage of metabolites commonly observed in metabolomic experiments in Spectraverse, MassSpecGym, and NPLIB1 was evaluated by performing reference spectral library searches with each of these three libraries against a data set of 29.1 million MS/MS spectra that we recently compiled from 4510 published metabolomic analyses of human blood.^54^ Cosine similarities were calculated using the implementation in matchms with a precursor *m*/*z* tolerance of 10 ppm and the requirement that at least three fragment ions matched between experimental and reference spectra.

## Results

### Comprehensive Curation and Harmonization of Reference MS/MS Spectra

Publicly available reference MS/MS spectra for small molecules are scattered across disparate sources. Databases such as GNPS and MoNA provide important resources for the computational mass spectrometry community by aggregating spectral libraries contributed by third parties.^4,55^ However, a substantial number of reference MS/MS spectra have not been integrated into these collections and instead are accessible only through repositories for raw metabolomics data such as MetaboLights,^46^ generic scientific data repositories such as Zenodo, or the supporting files or websites accompanying the publications that describe these libraries. Moreover, because databases such as GNPS and MoNA aggregate spectral libraries submitted by third parties, the MS/MS spectra therein have generally not been subjected to the same degree of curation as those found in commercial libraries. As a result, these databases can contain spectra that are of low quality, missing important pieces of metadata, or have chemical structure annotations that are inconsistent with the spectra themselves.^31,48^ To address these issues, we undertook an effort to comprehensively identify and curate publicly available small-molecule MS/MS spectra.

We began by retrieving all of the reference MS/MS spectra that we could identify. In addition to major databases and community resources (including GNPS, MoNA, MS-DIAL,^33^ HMDB,^34^ and MSnLib^35^), we curated data from individual publications and extracted reference spectra from raw LC–MS/MS data (Methods section). For example, we retrieved a reference MS/MS library for polyethylene-derived compounds from Zenodo,^37^ a library for *Caenorhabditis elegans* metabolites provided through an interactive web application,^41^ and a library of *Solanum lycopersicum* metabolites for which raw experimental data from reference standards was uploaded to MetaboLights,^44^ none of which had been deposited to community databases. In total, these efforts culminated in the accumulation of 1,672,217 small-molecule MS/MS spectra (Figure S1).

The completeness and coherence of the metadata associated with reference MS/MS spectra have come under increasing scrutiny in recent years. In turn, this scrutiny has spurred the development of tools such as matchms to harmonize spectral metadata and identify incoherent annotations.^31^ We therefore first leveraged matchms to harmonize the nomenclature of key metadata fields, derive missing metadata, and repair incorrect annotations that could be automatically identified and corrected (Figure 2a). In parallel, detailed examination of the MS/MS spectra that we had assembled led to the identification of a series of issues affecting the spectra themselves, including spectra for which all fragment ion intensities were identical; fragment ions with zero intensities; spectra acquired at MS^n^ levels of 3 or higher; spectra that were structurally uninformative (for instance, because the *m*/*z* values of all fragment ions were greater than the precursor *m*/*z*); and low-resolution spectra (Figure 2b–f). We devised a series of additional preprocessing steps to identify and repair or remove these problematic spectra (Methods section).

**Figure 2:**
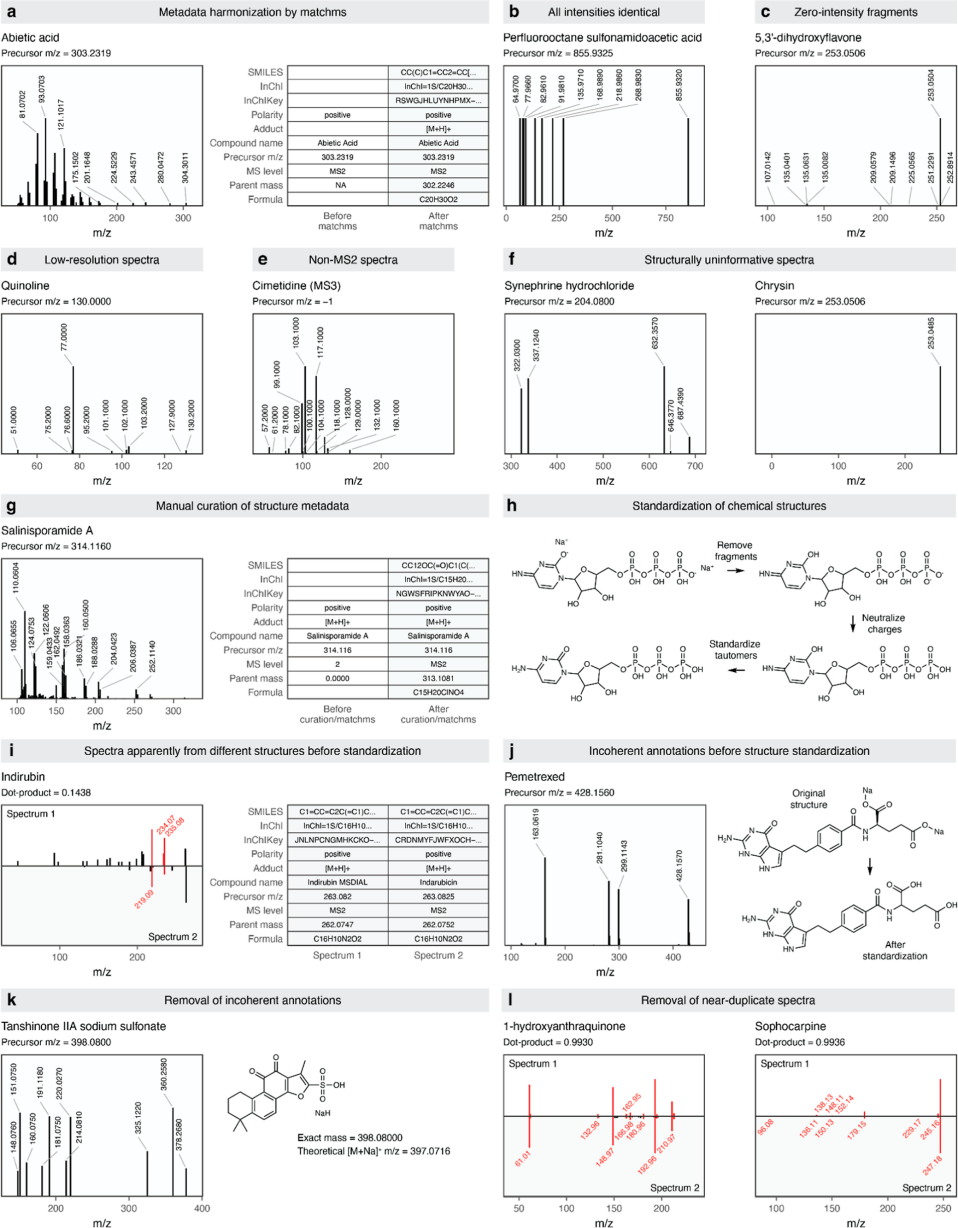
Representative examples for major categories of preprocessing steps in Spectraverse. (a) Harmonization of key metadata fields, derivation of missing metadata, and repair of incorrect metadata (when possible) with matchms. (b–g) Examples of categories of spectra removed or repaired during preprocessing. (b) Pseudo-MS/MS spectra with all fragment ions having identical intensities. (c) Spectra containing zero-intensity fragments. (d) Low-resolution MS/MS spectra. (e) Spectra acquired at MS^n^ levels beyond MS2. (f) Structurally uninformative spectra. Left, a spectrum where all fragment ion *m*/*z* values are greater than the precursor *m*/*z*. Right, a spectrum with a single fragment ion whose *m*/*z* lies within ±1.6 of the precursor *m*/*z*. (g) Example of a spectrum for which manual curation of missing structural metadata was performed, as this could not be automatically retrieved by matchms. (h) Key steps in the standardization of chemical structures associated with MS/MS spectra in Spectraverse. (i) A pair of spectra that appear to be associated with different structures (as determined by the first 14 characters of the InChIKey) before standardization but not after. Metadata is shown prior to standardization. (j) Example of a spectrum that is discarded by matchms when the associated structure is not first standardized using our pipeline. (k) Example of an incoherently annotated spectrum discarded by matchms; the precursor *m*/*z* is equal to the monoisotopic mass of the uncharged structure shown at the right. (l) Examples of near-duplicate spectra. Left, two spectra that appear to have been processed slightly differently, with the removal of low-intensity fragment ions in spectrum 1 but not in spectrum 2. Right, two spectra with very similar but not identical fragment ion intensities. See also the additional examples presented in Figure S2.

We then turned our attention to the chemical structures associated with each spectrum. Our treatment of these structures proceeded in three steps, each addressing a different challenge. First, we found that many spectra were associated with the common names of the corresponding small molecules but not a SMILES or InChI identifier and that matchms failed to retrieve the relevant structure for a considerable proportion of these spectra. We therefore implemented additional preprocessing routines to automatically retrieve these missing chemical structures and manually curated SMILES strings for 19,574 of the remaining spectra (corresponding to 2090 unique compounds; Figure 2g). Spectra for which a valid chemical structure could not be retrieved or which were annotated with invalid SMILES strings were removed. Second, we devised a pipeline to standardize the representations of each chemical structure, encompassing the removal of stereochemical information, neutralization of charged moieties, removal of disconnected fragments, and standardization of tautomers (Figure 2h). We found that particular care was needed to simultaneously standardize both the formal charges on the chemical structures and the adducts associated with the corresponding MS/MS spectra (Figures 2i,j and S2). For instance, we observed that neutralization of a single charged group within a zwitterion could cause matchms to reject the spectra associated with these molecules when they were annotated as [M + H]^+^ adducts because the resulting structure now carried a formal charge (Figure S2c). Conversely, other structures were represented in charged forms because the corresponding SMILES strings incorporated the adducts for which MS/MS spectra had been acquired (e.g., including a sodium cation for [M + Na]^+^ spectra; Figure S2e), thus requiring neutralization of the structure, removal of the adduct from the structure, and repair of the adduct metadata field in tandem. Third, we removed rare adducts for which it seemed unlikely that enough data were available to realistically support machine-learning models for MS/MS interpretation as well as adducts involving neutral losses, on the basis that it was generally unclear exactly which molecular species (or combination of species) were actually measured.

Having thus standardized the metadata associated with each reference MS/MS spectrum, we then applied matchms a second time, this time employing more stringent filters, to remove any remaining spectra with incoherent annotations (Figure 2k). We manually reviewed several thousand of the removed spectra to confirm that their associated metadata could not possibly have been repaired and made refinements to our preprocessing strategy that culminated in the workflow described above and in the Methods section. At this stage, we additionally removed any MS/MS spectra for which the observed precursor *m*/*z* deviated from the theoretical value by more than 10 ppm and MS/MS spectra collected on triple quadrupole mass spectrometers, both of which were assumed to denote low-resolution spectra.

A final series of filters ensured both the uniqueness of each MS/MS spectrum and the completeness of the associated metadata. In particular, we observed that spectral libraries contained large numbers of near-duplicate spectra, which might reflect the acquisition of virtually identical spectra with negligible differences in fragment ion intensity or the application of slightly different preprocessing strategies to the same MS/MS spectra (Figure 2l). To identify and filter these near-duplicate spectra, we kept only a single representative spectrum when multiple spectra from the same compound demonstrated a cosine similarity greater than 0.99. We experimented with various strategies for denoising spectra or removing low-intensity ions but found that these generally did not improve (and in fact slightly degraded) the propensity for spectral similarity to differentiate identical versus isobaric compounds within our data (Figure S3). Instead, we elected to filter very low-intensity fragment ions (with relative intensities less than 0.1% of the base peak) and retain only the top-4096 highest intensity ions per spectrum. Last, we manually curated and harmonized metadata associated with the type of mass spectrometer and collision energy at which each MS/MS spectrum had been acquired. To accommodate ramped or stepped collision energies, each spectrum was associated with up to three collision energy fields, each of which is provided both in eV and as a NCE within the spectrum metadata.

A total of 488,630 MS/MS spectra, spanning 44,237 unique small molecules, passed all of the above criteria, creating a comprehensive reference library of publicly available MS/MS spectra that we named Spectraverse.

## Spectraverse Expands on Existing Public Collections of Reference MS/MS Spectra

Our extensive efforts to curate and preprocess publicly available MS/MS spectra addressed numerous issues related to the quality of public MS/MS spectra and the coherence of their associated metadata. Some of these issues do not appear to have been previously documented, raising the possibility that they may have confounded reported differences in performance between machine-learning models for MS/MS interpretation or conversely have led valid MS/MS spectra to have been discarded from the training sets of these models. Even after such stringent filtering, however, Spectraverse represents the largest collection of publicly available reference MS/MS spectra. It contains more than twice as many spectra as MassSpecGym^48^ and more than 40 times as many as NPLIB1,^20^ the two primary noncommercial data sets that have been used to train and evaluate machine-learning models^17,18,20,23–25,30^ (Figure 3a). Moreover, we found that a considerable proportion of the spectra contained in MassSpecGym (*n* = 77,774) are near-duplicates, defined as spectra with a cosine similarity of 0.99 or greater to another spectrum from the same compound in the data set. After the removal of these near-duplicates, the increased scale of Spectraverse is even more apparent (Figure 3b).

**Figure 3:**
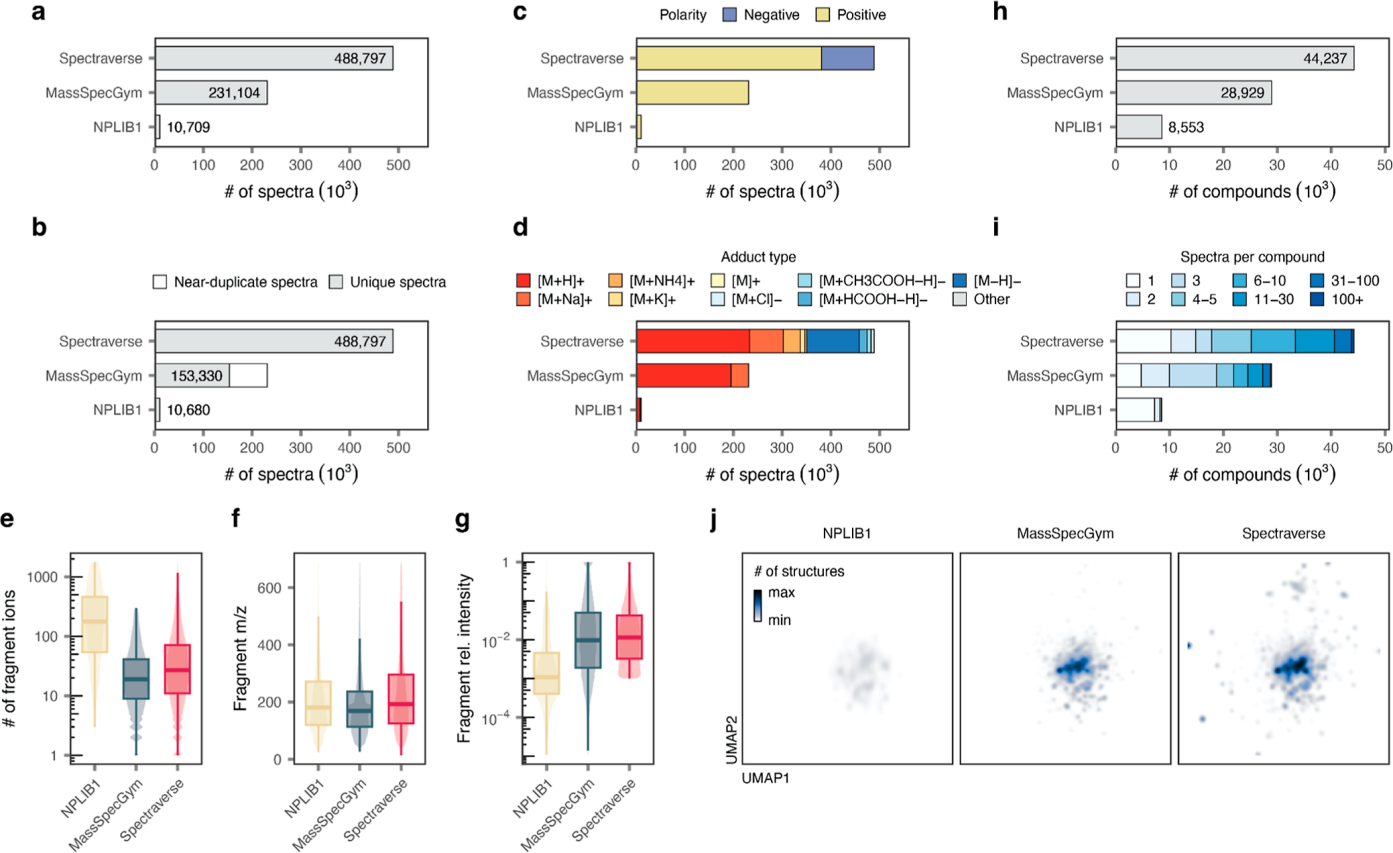
Overview of Spectraverse and comparison to MassSpecGym and NPLIB1. (a) Total number of MS/MS spectra in each data set. (b) As in (a) but after the removal of near-duplicate spectra (defined as spectra from the same compound, acquired in the same polarity, with a mutual cosine similarity greater than 0.99). (c) Number of MS/MS spectra per polarity. (d) Number of MS/MS spectra per adduct type. (e) Distribution of the number of fragment ions per spectrum in each data set. (f,g) Distribution of fragment *m*/*z* values (f) and relative intensities (g) in each data set. (h) Total number of structures represented by at least one MS/MS spectrum in each data set. (i) As in (h) but showing the number of spectra for each structure. (j) UMAP visualization of the structures encompassed by each data set. Fill reflects the number of structures at each coordinate.

Importantly, Spectraverse encompasses MS/MS spectra collected across a wide range of adducts commonly encountered in metabolomic experiments, many of which are absent or under-represented in MassSpecGym and NPLIB1 (Figure 3c,d). Whereas MassSpecGym in particular represents a highly valuable effort to standardize the benchmarking of computational methods for MS/MS interpretation, it contains only a fraction of all publicly available MS/MS spectra, in part because it excludes negative ionization mode spectra altogether as well as positive mode spectra beyond protonated or sodiated adducts.^48^ Flexibility in the computational interpretation of such spectra is essential to avoid misattributing them to the chemical “dark matter” of the metabolome by classifying them as signals arising from putatively unrecognized metabolites.^56^

Inspecting the characteristics of spectra contained within these three data sets—in particular, the number of fragment ions and their mass-to-charge ratios and intensities—also highlights an important advantage of both Spectraverse and MassSpecGym over NPLIB1 (Figures 3e–g and S4). In addition to its limited size, the latter data set contains spectra with an unusually large number of fragment ions, particularly low-intensity ions. This discrepancy likely reflects the inclusion of merged spectra, whereby scans acquired at various collision energies are consolidated to create a single representative spectrum.^20^ As Figure 3e and g underscore, such merged spectra may not resemble individual spectra acquired in routine metabolomic experiments.

We also sought to evaluate the chemical diversity of these three data sets, in addition to their technical heterogeneity (Figure 3h–j). To this end, we subjected the chemical structures found in all three data sets to dimensionality reduction using UMAP^57^ (Figure 3j). The resulting visualization highlights the broader coverage of chemical space afforded by Spectraverse.

Finally, we analyzed whether the technical heterogeneity and chemical diversity of Spectraverse translated into improved coverage of the metabolites encountered in routine metabolomic experiments. To assess this possibility, we leveraged a recently compiled data set of 29.1 million MS/MS spectra extracted from 4510 metabolomic analyses of human blood.^54^ We performed reference spectral library searches of these 29.1 million experimental MS/MS spectra against Spectraverse, MassSpecGym, or NPLIB1, using a 10 ppm precursor tolerance. Searching against Spectraverse enabled the annotation of the greatest number of experimental MS/MS spectra at any cosine similarity threshold (Figure 4).

**Figure 4:**
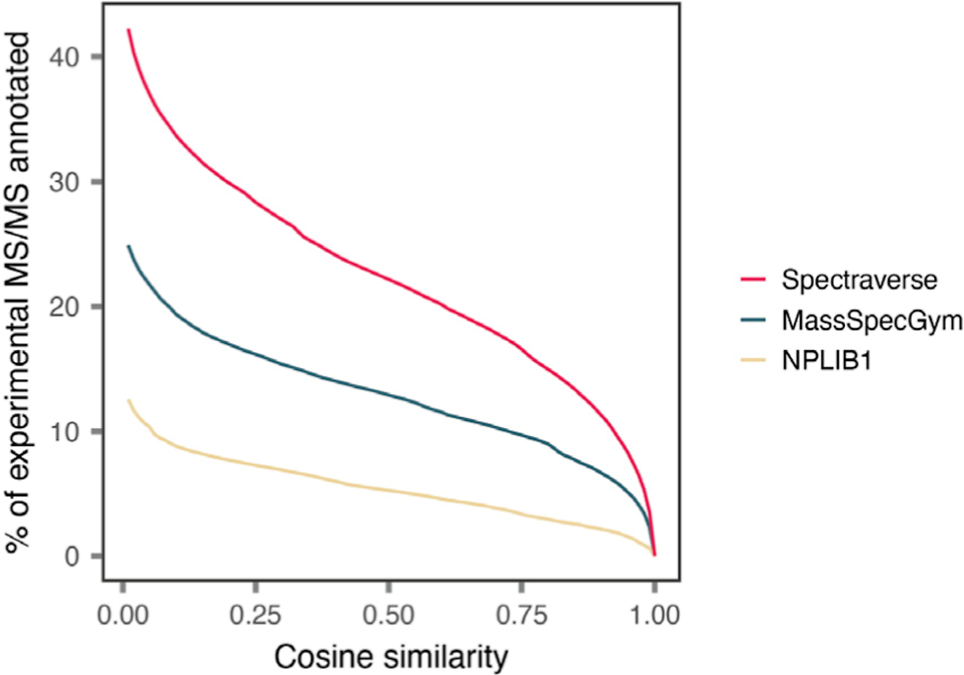
Cumulative distribution of cosine similarities between 29.1 million experimental MS/MS spectra extracted from published metabolomic analyses of human blood and reference MS/MS spectra in Spectraverse, MassSpecGym, or NPLIB1. The greatest number of experimental MS/MS spectra are annotated (at any cosine similarity threshold) when searching against Spectraverse.

## Discussion

Publicly available libraries of reference MS/MS spectra remain scattered across disparate sources and contain spectra that are of low quality, redundant, or accompanied by incomplete or incoherent metadata. As a result, investigators interested in developing machine-learning models for metabolite annotation must first undertake extensive efforts to curate, preprocess, and harmonize the reference spectra on which these models are to be trained. Such efforts require considerable expertise in small-molecule mass spectrometry, effectively raising the barrier to entry into this field.

Spectraverse addresses this barrier by assembling the largest and most diverse set of public MS/MS spectra to date, encompassing both positive and negative ionization modes and a broad spectrum of adduct types. It contains more than twice as many spectra as MassSpecGym, the largest existing resource of machine-learning-ready MS/MS spectra (and more than three times as many after removing near-duplicate spectra from this resource), and contains reference spectra for approximately 50% more small molecules. This breadth is notable given the stringent criteria that were applied to preprocess the MS/MS spectra therein. By manually reviewing several thousand MS/MS spectra, we identified and resolved a series of pitfalls that have not been consistently addressed in existing resources: for instance, the presence of low-resolution spectra, fragment ions with zero or uniform intensities, or inconsistent representations of chemical structures. The presence of tens of thousands of near-duplicate spectra in existing data sets is of particular concern, as it raises the possibility that models trained on such data may be inadvertently biased toward compounds or fragmentation patterns that are artifactually over-represented in these data sets.

The current release of Spectraverse (v1.0.1) is archived on Zenodo (https://doi.org/10.5281/zenodo.17870921), and our preprocessing code is publicly available via GitHub (https://github.com/skinniderlab/spectraverse-analysis), accompanied by extensive documentation. We intend to maintain and expand this resource in tandem with the continued growth of public MS/MS libraries. In particular, we plan to distribute future releases via Zenodo following a semantic versioning scheme such that each release will be assigned its own DOI and will be permanently archived, allowing interested parties to access old versions of Spectraverse (for instance, to reproduce the performance of a machine-learning model trained on a previous release). By doing so, we hope to ensure that machine-learning models for MS/MS interpretation are trained on the most accurate, diverse, and reproducible foundation possible and to lower the barriers for investigators who wish to leverage public MS/MS libraries in their own work.

## Supplementary Material



## Notes

Additional details relating to the creation and composition of Spectraverse, including examples of chemical structure standardization, impact of spectrum preprocessing, and additional comparisons to MassSpecGym and NPLIB1 and supplementary methods (PDF)
