# Supplementary material for: Comprehensive Curation and Harmonization of Small-Molecule MS/MS Libraries in Spectraverse
Source: Anal Chem. 2026 Jan 26;98(5):3934–43. doi: 10.1021/acs.analchem.5c06256 (PMC12903054; doi:10.1021/acs.analchem.5c06256)
Supplement: Supplementary file 1 [file ac5c06256_si_001.pdf]

Comprehensive curation and harmonization of small molecule MS/MS libraries in Spectraverse

Vishu Gupta[1,2], Hantao Qiang[1,2,3], Hsin-Hsiang Chung[1,2], Ehud Herbst[1,2], Michael A. Skinnider[1,2]*

[1] Lewis-Sigler Institute for Integrative Genomics, Princeton University, Princeton, NJ, 08544, USA
[2] Ludwig Institute for Cancer Research, Princeton University, Princeton, NJ, 08544, USA
[3] Department of Chemistry, Princeton University, Princeton, NJ, 08544, USA
* Correspondence: skinnider@princeton.edu

Supporting Information

Contents:

- S2-S6, Figures S1 to S4
- S7-S11, Supplementary Methods

Supplementary Figures

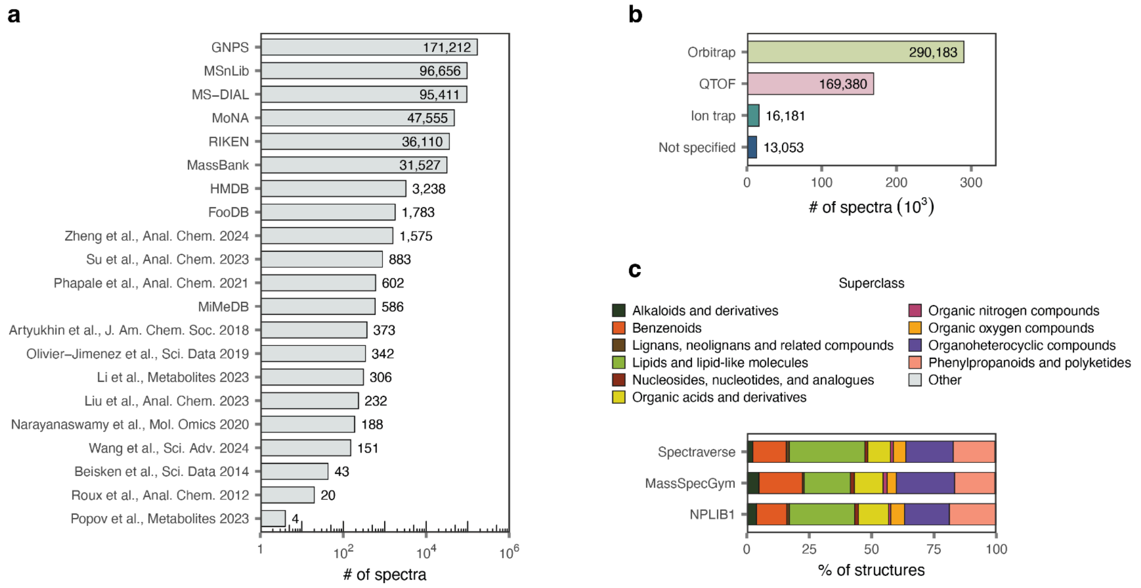

Figure S1. (a) Sources of the MS/MS spectra in Spectraverse. For duplicate or near-duplicate spectra that were present in multiple datasets or repositories, only the source associated with the retained spectrum is shown. (b) Instrument types associated with the MS/MS spectra in Spectraverse. (c) Distribution of ClassyFire superclasses for the small molecules in Spectraverse, MassSpecGym, and NPLIB1.

a Simultaneous standardization of structure and adduct

Cyanidine-3-O-sambubioside

Precursor m/z = 579.1350

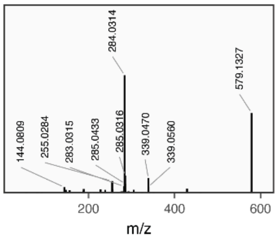

	Before standardization	After standardization
SMILES	OCC1OC(OC2=C([O...	O=C1C=C2OC(c3cc...
InChI	InChI=1S/C26H28...	InChI=1S/C26H28...
InChIKey	ZPPQIOUITZSYAO-...	DHNDHDGLZBNSFC-...
Polarity	negative	negative
Adduct	[M−2H]−	[M−H]−
Compound name	Cyanidine-3-O-sambubioside	Cyanidine-3-O-sambubioside
Precursor m/z	579.135	579.135
MS level	MS2	MS2
Parent mass	580.1428	580.1428
Formula	C26H27O15−	C26H28O15

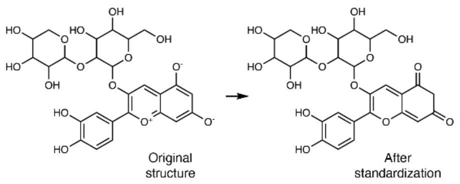

b Spectra apparently from different structures before standardization

Balsalazide

Dot-product = 0.0000

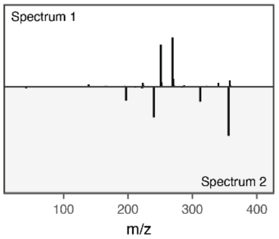

	Spectrum 1	Spectrum 2
SMILES	OC(=O)CCNC(=O)c...	C1=CC(=CC=C1C(=...
InChI	InChI=1S/C17H15...	InChI=1S/C17H15...
InChIKey	IPOKCKJONYRRHP-...	KONZVQJABTUMFX-...
Polarity	positive	negative
Adduct	[M+H]+	[M−H]−
Compound name	nan	Balsalazide
Precursor m/z	358.103	356.0875
MS level	2	MS2
Parent mass	357.0957	357.0948
Formula	C17H15N3O6	C17H15N3O6

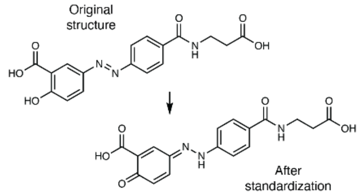

c Prevent charge neutralization for consistency with adduct

Butyryl-carnitine

Precursor m/z = 232.1549

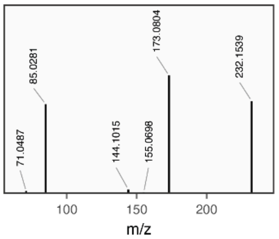

	Before standardization	After standardization
SMILES	CCCC(=O)O[C@H](...	CCCC(=O)OC(CC(=...
InChI	InChI=1S/C11H21...	InChI=1S/C11H21...
InChIKey	QWYFHHGCZUCMBN-...	QWYFHHGCZUCMBN-...
Polarity	positive	positive
Adduct	[M+H]+	[M+H]+
Compound name	R−Butyryl carnitine	R−Butyryl carnitine
Precursor m/z	232.154883152	232.154883152
MS level	MS2	MS2
Parent mass	231.1476	231.1476
Formula	C11H21NO4	C26H28O15

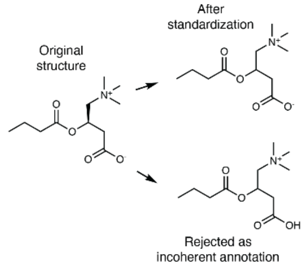

d Charge neutralization required for consistency with adduct

Unnamed compound

Precursor m/z = 501.3400

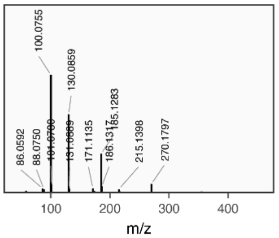

	Before standardization	After standardization
SMILES	CN(C(C)=O)CCN(C...	CC(=O)N(C)CCN(C...
InChI	InChI=1S/C23H44...	InChI=1S/C23H44...
InChIKey	GLTJQMHLSPIFLN-...	GLTJQMHLSPIFLN-...
Polarity	positive	positive
Adduct	[M+H]+	[M+H]+
Compound name		
Precursor m/z	501.34	501.34
MS level	2	MS2
Parent mass	500.3327	500.3322
Formula	C23H45N6O6+	C23H44N6O6

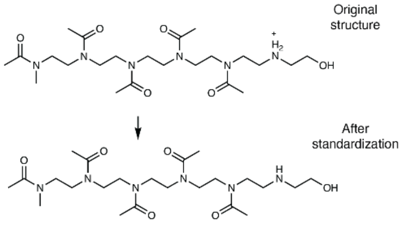

e Adduct written into SMILES

Unnamed compound

Precursor m/z = 466.2640

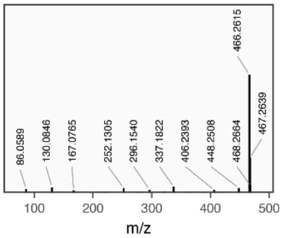

	Before standardization	After standardization
SMILES	O=C(C)NCCN(C(C)...	CC(=O)NCCN(CCN(...
InChI	InChI=1S/C20H37...	InChI=1S/C20H37...
InChIKey	HJWBHNWDFJBGNS-...	AEWYTTFQCXKWQF-...
Polarity	positive	positive
Adduct	[M+H]+	[M+Na]+
Compound name		
Precursor m/z	466.264	466.264
MS level	2	MS2
Parent mass	465.2563	443.2744
Formula	C20H37N5NaO6+	C20H37N5O6

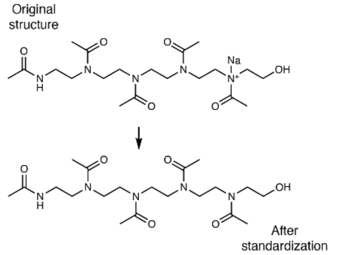

Figure S2. Additional representative examples highlighting the need for careful standardization of chemical structures in Spectraverse. (a) An example of a structure for which both the structure and adduct must be repaired in tandem. The original structure has a charge of –1 and is annotated as a $[M-2H]^{-}$ adduct. After standardization, the structure has no charge and is annotated as a $[M-H]^{-}$ adduct. (b) A second example of a pair of spectra that appear to be associated with different structures (as determined by the first fourteen characters of the InChIKey) before standardization, but not after. (c) An example of a structure for which neutralization of one charged group (without concomitant adjustment of the adduct) results in an apparently incoherent annotation that will be rejected by matchms. Preserving the zwitterionic form of the structure results in a valid annotation. (d) An example of a structure for which neutralization of the charged form is required for a valid annotation (i.e., one which is not discarded by matchms). (e) An example of a structure requiring particularly careful standardization of the structure and adduct in tandem because the adduct has been incorporated into the SMILES string itself. The original structure contains a sodium ion, has a formal charge, and is annotated as a $[M+H]^{+}$ adduct. Our pipeline removes the sodium ion, neutralizes the charge, and recodes the adduct as $[M+Na]^{+}$.

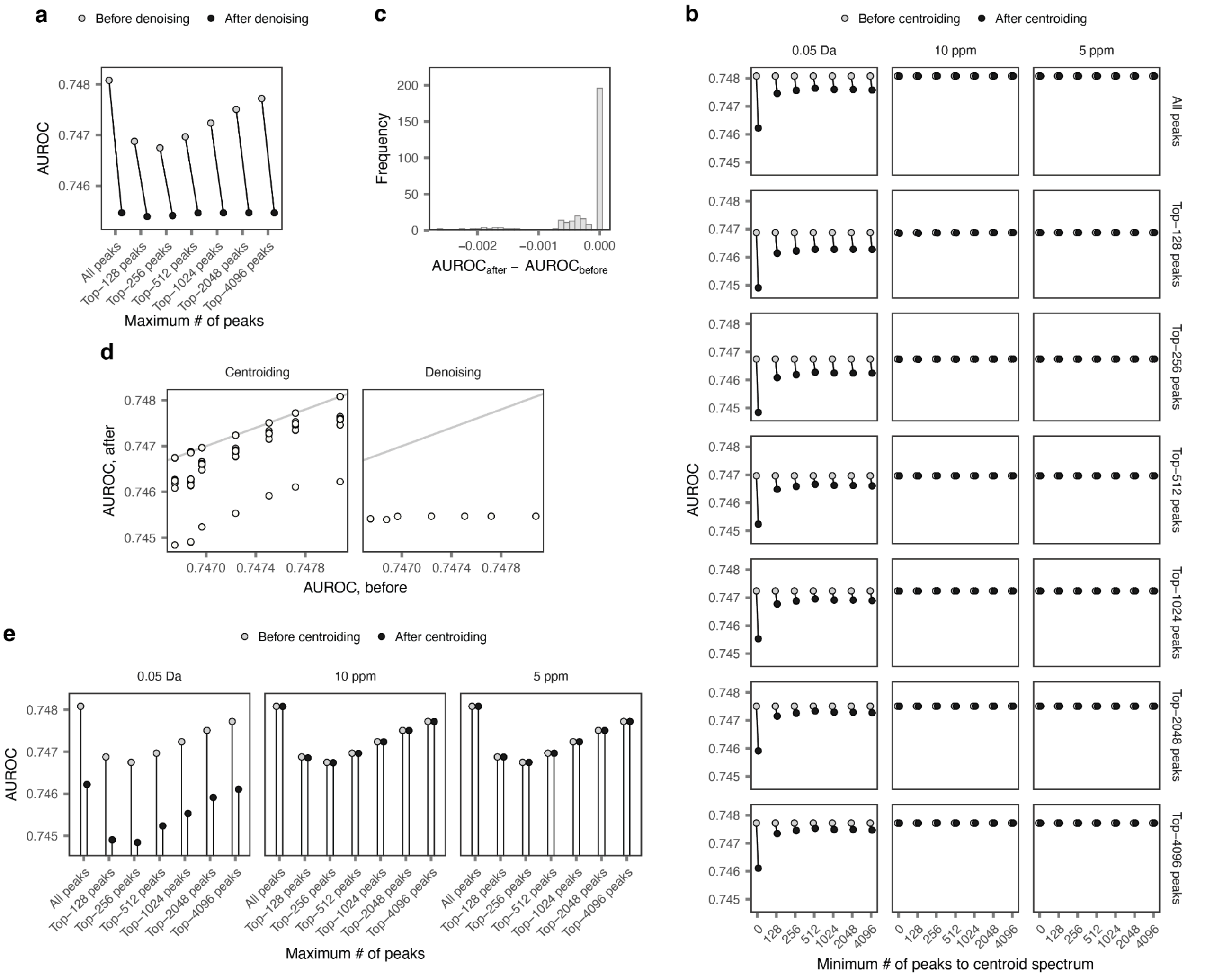

Figure S3. Impact of spectrum preprocessing by centroiding, electronic denoising, or removal of low-intensity peaks. All analyses show the propensity for spectral similarity (as quantified by the cosine similarity) to correctly differentiate spectra from the same versus isobaric compounds, as quantified by the area under the receiver operating characteristic curve (AUROC). (a) Impact of electronic denoising[47], shown when filtering spectra to retain only the top-*n* highest intensity fragment ions or when retaining all ions. (b) Impact of centroiding, shown when filtering spectra to retain only the top-*n* highest intensity fragment ions or when retaining all ions, and also when requiring spectra to contain a minimum number of fragment ions in order to apply the centroiding function. (c) Histogram of differences in the AUROC before versus after centroiding or electronic denoising. Both preprocessing functions consistently decrease the AUROC. (d) As in (c) but shown as a scatterplot, separately for centroiding versus electronic denoising. (e) As in (b), but shown as a function of the maximum number of fragment ions retained, without requiring a minimum number of fragment ions to apply centroiding.

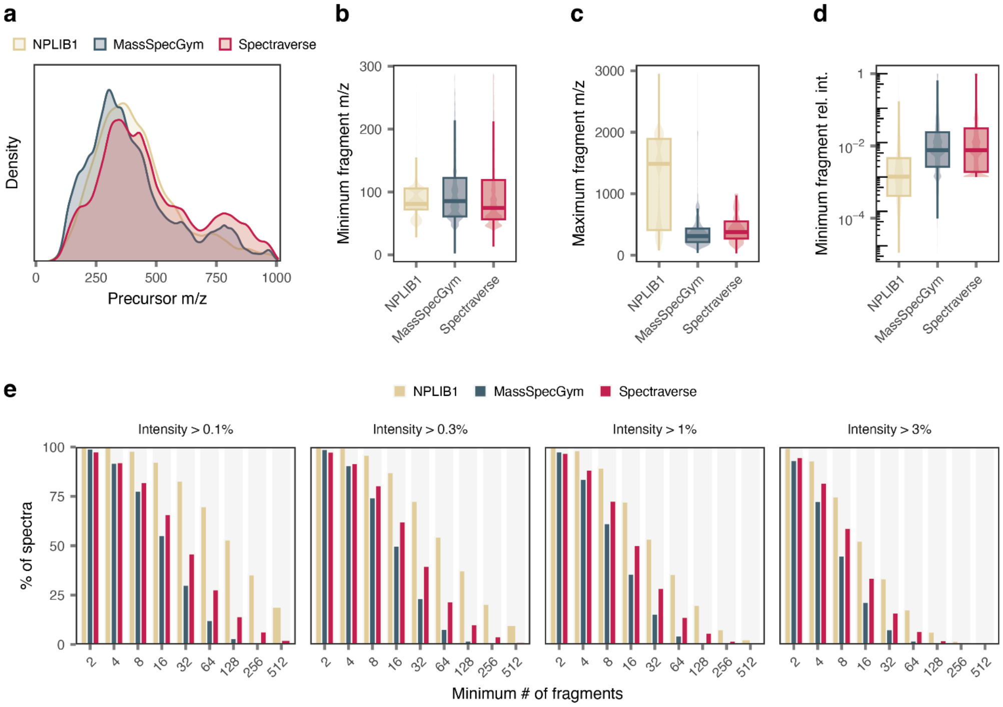

Figure S4. Additional comparisons of Spectraverse to MassSpecGym and NPLIB1. (a) Distribution of precursor m/z values in each dataset. (b-c) Distribution of minimum (b) and maximum (c) fragment m/z values per spectrum in each dataset. (d) Distribution of minimum relative fragment intensities (scaled to the base peak) in each dataset. (e) Proportion of spectra in each dataset that contain at least *n* fragments (x-axis) above a given intensity threshold, relative to the base peak.

Supplementary Methods

Data collection

GNPS

An aggregated file containing reference MS/MS libraries hosted by the GNPS database[55] was obtained from the GNPS website (https://external.gnps2.org/gnpslibrary, file: matchms.mgf) in MGF format on September 18, 2024. Spectra contained within the DEREPLICATOR_IDENTIFIED_LIBRARY library were removed, on the basis that structures had been assigned *in silico*[58]. Adduct metadata was manually extracted from the NAME metadata field, when missing. Other metadata field names were manually standardized. Spectra that were not associated with a compound name were retrieved by querying the GNPS API using the SPECTRUMID, when available.

MoNA

An aggregated file containing all reference LC-MS/MS spectra available in the MoNA database was obtained from the MoNA website (https://mona.fiehnlab.ucdavis.edu/downloads) in MSP format on November 18, 2023, and converted to MGF format. No further preprocessing was applied.

MassBank

An aggregated file containing all reference LC-MS/MS spectra available in the MassBank database was obtained from the MassBank-data GitHub repository (https://github.com/MassBank/MassBank-data, release 2024.06) in MSP format and converted to MGF format. No further preprocessing was applied.

MS-DIAL

A total of 25 reference MS/MS libraries distributed by the developers of MS-DIAL[33] were obtained from the MS-DIAL project website (https://systemsomicslab.github.io/compms/msdial/main.html) in MSP format on October 26, 2023. MSP files were converted to MGF format, and metadata field names were manually standardized. Spectra not associated with a SMILES string were removed.

RIKEN

A series of reference MS/MS libraries were obtained from the RIKEN website (https://www.riken.jp/) on March 18, 2024. First, the ReSpect library of phytochemicals[59] was obtained from its current location at ttp://prime.psc.riken.jp/menta.cgi/prime/legacy_index#PL0003 in .txt format. These files were converted to MGF format using custom Python code, and metadata fields were manually standardized.
Only high-resolution MS/MS spectra (those containing m/z values recorded to more than two decimal places) were retained. SMILES strings were retrieved by using the PubChem Identifier Exchange Service to convert the CAS numbers associated with each spectrum. Second, a reference MS/MS library for lipidomics was obtained from https://metabography.riken.jp/menta.cgi/lipidomics/download_data_set in MGF format. Ionization modes were parsed manually based on the annotated adducts and metadata fields were manually standardized. Only spectra associated with a valid SMILES string were retained. Third, a MS/MS library for plant specialized metabolites was obtained from http://planmaxdb.riken.jp/menta.cgi/planmax/download/plant/v1/contents in MSP format and converted to MGF format. Ionization modes were parsed manually based on the annotated adducts and metadata fields were manually standardized. Only spectra associated with a valid SMILES string were retained.

HMDB[34]

A library of reference MS/MS spectra from human metabolites was obtained from the HMDB website in XML format (https://hmdb.ca/downloads, dated July 1, 2023). XML files were converted to MGF format using custom code and combined. Structure metadata (compound name, SMILES, InChI and InChIKey) was then added by querying an XML file also obtained from the HMDB website containing all metabolites present in this release (dated November 17, 2021). Only spectra for which a valid SMILES could be retrieved were retained.

FooDB

A library of reference MS/MS spectra for dietary metabolites was obtained from the FooDB website in XML format (https://foodb.ca/downloads, dated October 13, 2022). XML files were converted to MGF format using custom code and combined, extracting instrument type, ionization mode, collision energy (in eV), and adduct types from the XML files, renaming metadata fields when the relevant values were encoded in a different XML tag (e.g., extracting SMILES strings from the <cas_number> tag and InChIKeys from the <moldb_smiles> tag). Structure metadata (compound name, SMILES, InChI and InChIKey) was then added by querying a CSV file also obtained from the FooDB website (Compound.csv, dated April 7, 2020) containing all metabolites present in this release. Only spectra for which a valid SMILES could be retrieved, and which were associated with a discernible adduct type and precursor m/z, were retained. Only spectra for which a valid SMILES could be retrieved, and which were associated with a discernible adduct type and precursor m/z, were retained.

MiMeDB[60]

A library of reference MS/MS spectra for small molecule metabolites found in the human microbiome was obtained from the MiMeDB website in XML format (https://mimedb.org/downloads, dated October 17, 2022). XML files were converted to MGF format using custom code and combined, extracting instrument type, ionization mode, and collision energy (in eV) from the appropriate fields in the XML file and extracting adduct types from the <notes> field using regular expressions. Structure metadata (compound name, SMILES, InChI and InChIKey) was then added by querying a CSV file also obtained from the MiMEDB website (mimedb_metabolites_v1.csv) containing all metabolites present in this release (dated March 19, 2024). Only spectra for which a valid SMILES could be retrieved, and which were associated with a discernible adduct type and precursor m/z, were retained.

MSnLib[35]

Eight reference MS/MS libraries were obtained from the Zenodo accession accompanying the manuscript (https://zenodo.org/records/11163381; file names: 20231031_nihnp_library_neg_all_lib_MS2_processed.mgf, 20231031_nihnp_library_pos_all_lib_MS2_processed.mgf, 20231130_mcescaf_library_neg_all_lib_MS2_processed.mgf, 20231130_mcescaf_library_pos_all_lib_MS2_processed.mgf, 20231130_otavapep_library_neg_all_lib_MS2_processed.mgf, 20231130_otavapep_library_pos_all_lib_MS2_processed.mgf, 20240411_mcebio_library_neg_all_lib_MS2_processed.mgf, and 20240411_mcebio_library_pos_all_lib_MS2_processed.mgf) on September 3, 2024. Metadata fields were manually standardized.

Roux et al., *Anal. Chem.* 2012[45]

A reference MS/MS library for human urine metabolites was obtained from the supplementary tables accompanying the manuscript. Fragment ion m/z and intensity values were extracted and converted to MGF format, along with compound name and collision energy metadata. Compound names were then matched to structural identifiers (SMILES, InChI, and formula) using the metabolite identification file (m_MS2neg_FIAMS2_metid_urine_v2_maf.tsv) uploaded to the MetaboLights repository (accession MTBLS20).

Beisken et al., *Sci. Data* 2014[44]

A reference MS/MS library for *Solanum lycopersicum* metabolites was constructed by re-processing raw LC-MS/MS data from a series of reference standards, which was obtained from MetaboLights (MTBLS38). First, precursor m/z values, retention times, and structural metadata (SMILES, InChI, and formula) were obtained from the metabolite annotation table (m_MTBLS38_metabolite_standards_metabolite_profiling_mass_spectrometry_v2_maf.tsv). Filenames from standard injections were then matched to metabolite names stored in the 'metabolite_identification' column of the metabolite annotation table using a combination of fuzzy string matching and manual review. Then, MS/MS spectra corresponding to the metabolites of interest were extracted from these files, using a 20 ppm window around the theoretical precursor m/z and a 5 second window around the retention time noted in the metabolite

annotation file. These MS/MS spectra were then annotated with metadata stored in the metabolite annotation table. When more than one spectrum was obtained, they were merged using the function ‘combineSpectra’ in the Bioconductor package ‘Spectra’. Each merged spectrum was manually inspected by generating a mirror plot comparing the original and merged spectra to confirm the similarity of the merged spectra. Missing metadata (ionization mode, instrument type, collision energy) was manually assigned based on values reported in the manuscript, metadata field names were manually standardized, and the complete set of spectra was exported to MGF format.

Artyukhin et al., *J. Am. Chem. Soc.* 2018[41]
A reference MS/MS library for *C. elegans* metabolites was obtained from the SMID-DB website accompanying the publication on April 4, 2024. Compound pages were accessed in sequence to retrieve structural metadata (SMILES, IUPAC name, molecular formula, and any comments) as well as ionization mode, collision energy, and adduct type. Fragment m/z and intensity values were accessed from the linked spectrum pages. Spectra were then parsed and saved to MGF format using the ‘Spectra’ R package. Precursor m/z values were imputed based on the neutral mass of the structure encoded in SMILES format and the corresponding adduct, when missing. Spectra for which the reported adduct was inconsistent with the reported ionization mode were discarded. Moreover, the molecular formula was recalculated from the SMILES string using RDKit, and spectra for which the calculated and annotated formulas were inconsistent were manually reviewed and discarded if the inconsistency could not be resolved. Metadata field names were then manually standardized.

Olivier-Jimenez et al., *Sci. Data* 2019[61]
A reference MS/MS library for lichen metabolites was obtained from the MetaboLights repository (accession MTBLS999) in mzXML format. Ionization modes, compound names, and collision energies were parsed from the filenames, and the files were converted to MGF format. Additional structural metadata was then retrieved from the metabolite identification files in TSV format. Instrument type data was assigned based on the manuscript, and metadata fields were manually standardized.

Narayanaswamy et al., *Mol. Omics* 2020[39]
A reference MS/MS library for metabolites included in the Mass Spectrometry Metabolite Library (MSMLS) kit (Sigma-Aldrich) was obtained from MetaboLights (accession: MTBLS1311) in MSP format on November 29, 2023. The file was converted to MGF format, and SMILES strings were retrieved for each spectrum by using the PubChem Identifier Exchange Service to convert the InChIKeys associated with each spectrum. Missing metadata (ionization mode, instrument type, collision energy) was manually assigned based on values reported in the manuscript, and metadata fields names were manually standardized.

Phapale et al., *Anal. Chem.* 2021[62]
A reference MS/MS library for endogenous metabolites and lipids was obtained from the website accompanying the publication (https://curatr.mcf.embl.de/MS2/export/) in MGF format on November 21, 2023. Metadata in the accompanying TSV file was incorporated into the spectra, validating the alignment of precursor m/z values between both sources. The instrument type was manually curated from the accompanying publication and collision energies were standardized replacing phrases such as "collision energy X electronvolt" with "X eV". SMILES strings for each compound were retrieved by using the PubChem Identifier Exchange Service to convert the InChIKeys associated with each spectrum. Metadata fields were then manually standardized.

Liu et al., *Anal. Chem.* 2023[36]
A reference MS/MS library for pharmaceutical compounds and their impurities (“Drug+”) was obtained from the Zenodo accession accompanying the manuscript (https://zenodo.org/records/7531170) in MGF format on December 1, 2023. The dataset was filtered to retain only spectra explicitly annotated as originating from the in-house compound library (i.e., those with DB == "Inhouse"), as the remaining spectra were aggregated from public databases. Instrument type and collision energy was manually entered based on the values reported in the paper, and metadata field names were manually standardized.

Su et al., *Anal. Chem.* 2023[37]
A reference MS/MS library for 500 plastic-related chemicals was obtained from the GitHub repository accompanying the paper (https://github.com/QizhiSu/MS-libraries) in MGF format on October 5, 2023. Metadata field names were manually standardized.

Li et al., *Metabolites* 2023[42]
A reference MS/MS library for plant metabolites was obtained from the Zenodo accession accompanying the manuscript (https://zenodo.org/records/6916522) in MGF format on September 16, 2024, and converted to MGF format. Metadata field names were manually standardized.

Popov et al., *Metabolites* 2023[38]
A reference MS/MS library for sea cucumber triterpene glycosides was obtained from the supplementary material accompanying the paper in MGF format. Collision energies for each spectrum were manually inferred based on the number of sodium adducts present in the corresponding SMILES string and the statement in the manuscript that energies of 120, 60, 43, and 40 eV were used for precursor ion charges of 1, 2, 3, and 4, respectively, and metadata field names were manually standardized.

Wang et al., *Sci. Adv.* 2024[40]
Two reference MS/MS libraries for per- and polyfluoroalkyl substances (PFAS) were obtained from the Zenodo accession accompanying the manuscript (https://zenodo.org/records/10977307; files PFAS-identified.mgf and PFAS-library.mgf) in MGF format on September 5, 2024, and metadata field names were manually standardized.

Zheng et al., *Anal. Chem.* 2024[43]
A reference MS/MS library for gut microbiota-related metabolites was obtained from the supplementary tables accompanying the manuscript. Fragment ion m/z and intensity values were extracted and converted to MGF format, along with metadata (compound name, SMILES, InChIKey, formula polarity, adduct, instrument type, and collision energy).

Standardization of instrument types and collision energies
Instrument types were manually reviewed and standardized according to the dictionary below:

```
{'qtof': 'qtof', 'q-tof': 'qtof', 'qtfo': 'qtof', 'orbitrap': 'orbitrap', 'qqq': 'qqq', 'qq':
'qqq', 'ittof': 'iontrap', 'it-tof': 'iontrap', 'ion trap': 'iontrap', 'ion-trap': 'iontrap',
'tripletof': 'qtof', 'triple tof': 'qtof', 'qft': 'orbitrap', 'itft': 'orbitrap', 'hf':
'orbitrap', 'q exactive': 'orbitrap', 'q-exactive': 'orbitrap', 'ttof': 'qtof', 'LC-ESI-CID;
Velos': 'iontrap', 'LC-ESI-HCD Velos': 'orbitrap', 'Bruker timsTOF Pro': 'qtof',
'LC-ESI-QIT;4000Q TRAP': 'qtof', 'LC-ESI-TOF impact HD': 'qtof', 'LC-ESI-HCD Lumos':
'orbitrap', 'LC-ESI-CID; Lumos': 'iontrap', 'FTMS-ESI': 'orbitrap', 'Thermo Finnigan LTQ':
'iontrap', 'MALDI-TOFTOF JMS-S3000': 'qtof', 'ESI-TOF;micrOTOF-Q': 'qtof', 'ESI-HCD':
'orbitrap', 'impact HD': 'qtof', 'MALDI-TOFTOF': 'qtof', 'LC-ESI-TOF;LCT Micromass': 'qtof',
'FAB-EBEB': 'qtof', 'MALDI-QIT;AXIMA QIT': 'qtof', 'QIT;API QSTAR': 'qtof', 'Waters SYNAPT':
'qtof' }
```

Spectra associated with low-resolution triple quadrupole (QQQ) instruments were manually removed. Because we observed that initial versions of Spectraverse sometimes contained spectra that were not annotated with an instrument type, but which were virtually identical to QQQ spectra that were discarded in the final stage of the dataset preparation, spectra were sorted prior to deduplication so as to preferentially retain spectra acquired on QQQ instruments, so that they could be discarded later.

All unique values in the collision energy metadata field were likewise manually reviewed to determine how best to standardize these values. We found that collision energies (CE) were generally specified in electronvolts (eV) or as normalized collision energies (NCE), but not both. Therefore, when only one was specified, the missing counterpart was computed using the function

$$\mathrm{CE}(p) = \frac{\mathrm{m}(p) \times \mathrm{NCE}(p)}{500}$$

For entries associated with a single CE or NCE value, this value was assigned to the fields COLLISION_ENERGY_1 or NORMALIZED_COLLISION_ENERGY_1. For entries with ramped CE or NCE values, the minimum and maximum values were assigned to COLLISION_ENERGY_1 and COLLISION_ENERGY_2 (NORMALIZED_COLLISION_ENERGY_1 and NORMALIZED_COLLISION_ENERGY_2), respectively. For entries with stepped CE or NCE values, the minimum, intermediate, and maximum values were assigned to COLLISION_ENERGY_1, COLLISION_ENERGY_2, and COLLISION_ENERGY_3 (NORMALIZED_COLLISION_ENERGY_1, NORMALIZED_COLLISION_ENERGY_2 and NORMALIZED_COLLISION_ENERGY_3), respectively.

Supplementary References

(58) Mohimani, H.; Gurevich, A.; Shlemov, A.; Mikheenko, A.; Korobeynikov, A.; Cao, L.; Shcherbin, E.; Nothias, L.-F.; Dorrestein, P. C.; Pevzner, P. A. Dereplication of Microbial Metabolites through Database Search of Mass Spectra. *Nat. Commun.* **2018**, *9* (1), 4035.

(59) Sawada, Y.; Nakabayashi, R.; Yamada, Y.; Suzuki, M.; Sato, M.; Sakata, A.; Akiyama, K.; Sakurai, T.; Matsuda, F.; Aoki, T.; Hirai, M. Y.; Saito, K. RIKEN Tandem Mass Spectral Database (ReSpect) for Phytochemicals: A Plant-Specific MS/MS-Based Data Resource and Database. *Phytochemistry* **2012**, *82*, 38–45.

(60) Wishart, D. S.; Oler, E.; Peters, H.; Guo, A.; Girod, S.; Han, S.; Saha, S.; Lui, V. W.; LeVatte, M.; Gautam, V.; Kaddurah-Daouk, R.; Karu, N. Mimedb: The Human Microbial Metabolome Database. *Nucleic Acids Res.* **2023**, *51* (D1), D611–D620.

(61) Olivier-Jimenez, D.; Chollet-Krugler, M.; Rondeau, D.; Beniddir, M. A.; Ferron, S.; Delhaye, T.; Allard, P.-M.; Wolfender, J.-L.; Sipman, H. J. M.; Lücking, R.; Boustie, J.; Le Pogam, P. A Database of High-Resolution MS/MS Spectra for Lichen Metabolites. *Sci. Data* **2019**, *6* (1), 294.

(62) Phapale, P.; Palmer, A.; Gathungu, R. M.; Kale, D.; Brügger, B.; Alexandrov, T. Public LC-Orbitrap Tandem Mass Spectral Library for Metabolite Identification. *J. Proteome Res.* **2021**, *20* (4), 2089–2097.
